# Supplementary material for: Expression and role of melatonin membrane receptors in the hypothalamic-pituitary-testicular axis of Tibetan sheep in a plateau pastoral area
Source: PLoS One. 2023 Oct 25;18(10):e0290775. doi: 10.1371/journal.pone.0290775 (PMC10599587; doi:10.1371/journal.pone.0290775)
Supplement: S1 Raw images — (ZIP) [file pone.0290775.s001.zip › Supporting Information file/gels' original images.pdf]

Original Images for Gels

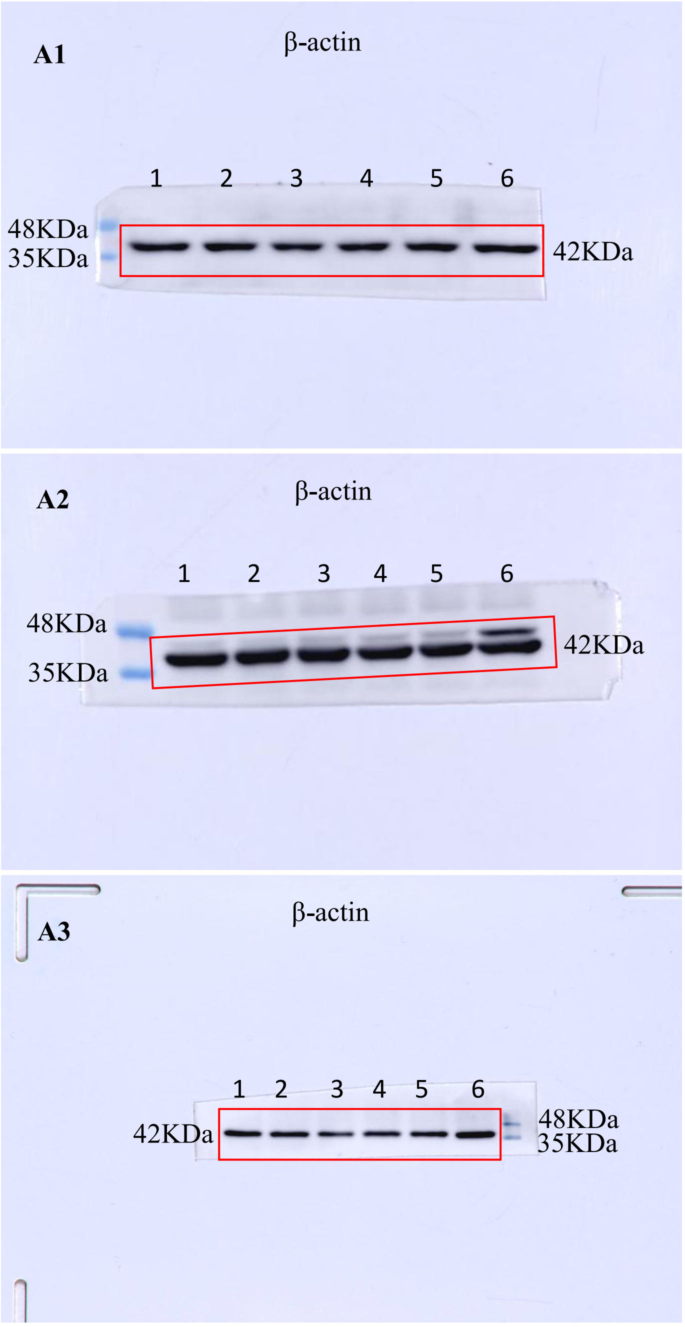

Figures A1, A2, and A3 are protein gel blots of three replicate experiments of Tibetan sheep β-actin. 1, 2, 3, 4, 5, 6 represent hypothalamus, hypophysis, testis, epididymal caput, epididymal corpus, and epididymal cauda, respectively. All Western blot gels are cropped with reference to the Marker prior to hybridization with the antibody. Gel strips are developed in a chemiluminescence instrument. The red box in the figure is the imprint of the target protein and the blue imprint is the Marker.

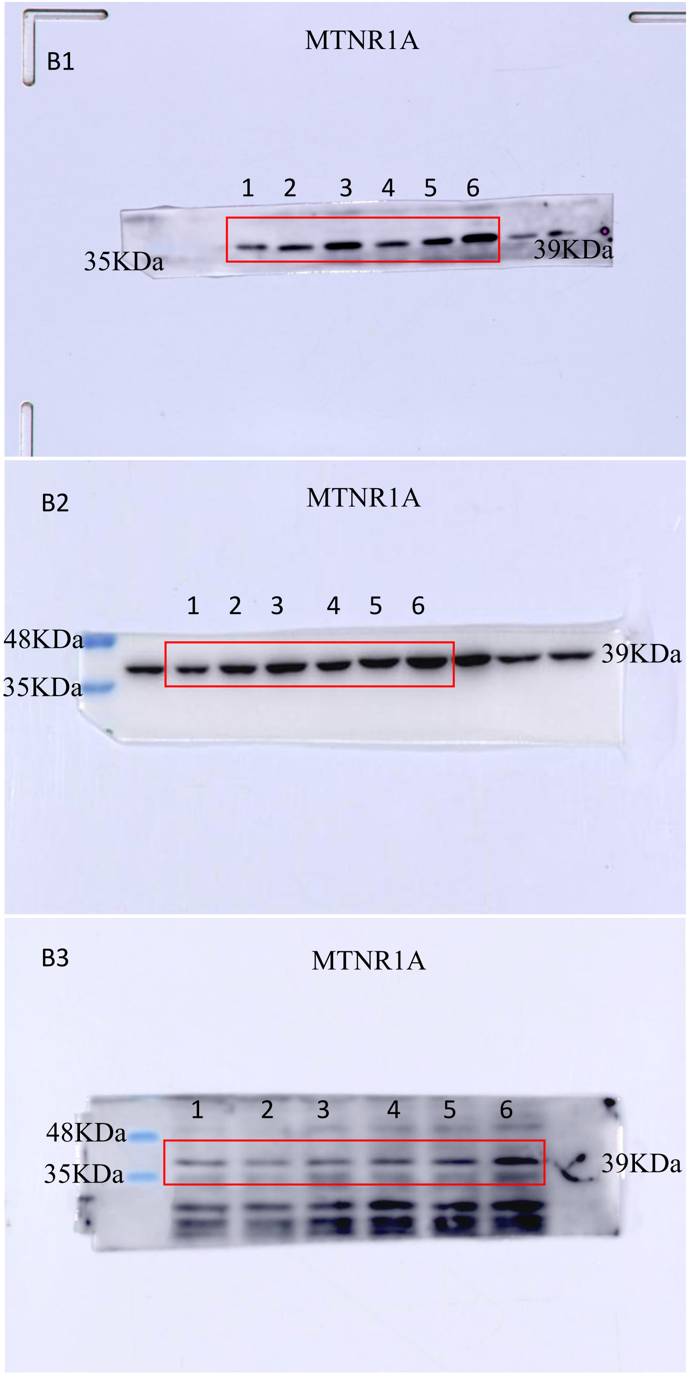

Figures B1, B2, and B3 are protein gel blots of three replicate experiments of Tibetan sheep MTNR1A. 1, 2, 3, 4, 5, 6 represent hypothalamus, hypophysis, testis, epididymal caput, epididymal corpus, and epididymal cauda, respectively. All Western blot gels are cropped with reference to the Marker prior to hybridization with the antibody. Gel strips are developed in a chemiluminescence instrument. The red box in the figure is the imprint of the target protein and the blue imprint is the Marker.

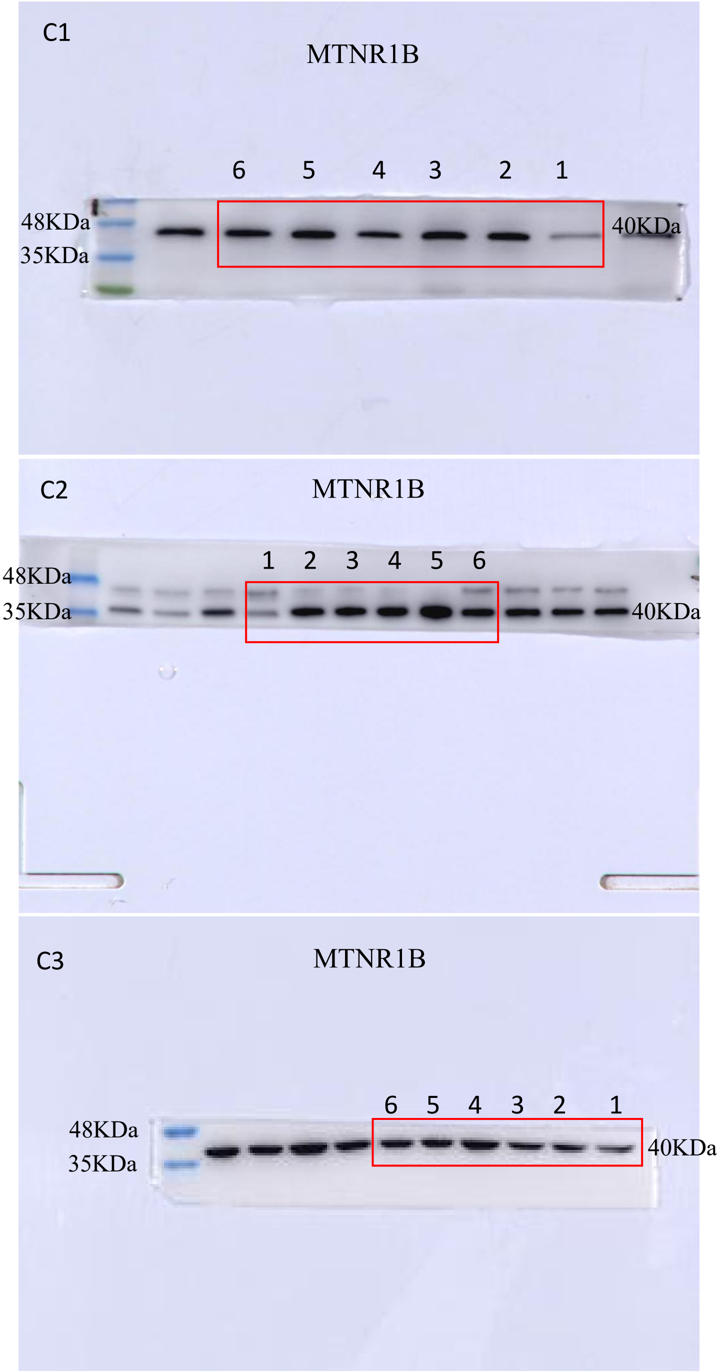

Figures C1, C2, and C3 are protein gel blots of three replicate experiments of Tibetan sheep MTNR1B. 1, 2, 3, 4, 5, 6 represent hypothalamus, hypophysis, testis, epididymal caput, epididymal corpus, and epididymal cauda, respectively. All Western blot gels are cropped with reference to the Marker prior to hybridization with the antibody. Gel strips are developed in a chemiluminescence instrument. The red box in the figure is the imprint of the target protein and the blue imprint is the Marker.

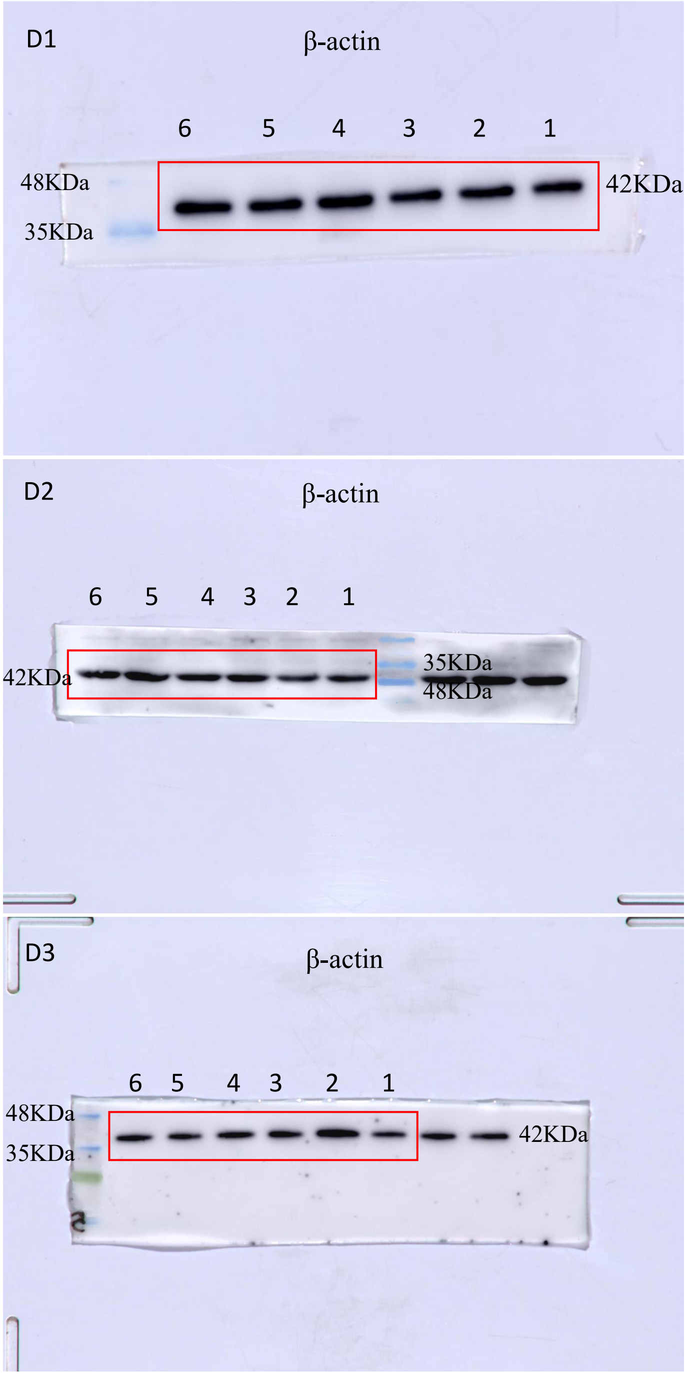

Figures D1, D2, and D3 are protein gel blots of three replicate experiments of Small Tail Han sheep β-actin. 1, 2, 3, 4, 5, 6 represent hypothalamus, hypophysis, testis, epididymal caput, epididymal corpus, and epididymal cauda, respectively. All Western blot gels are cropped with reference to the Marker prior to hybridization with the antibody. Gel strips are developed in a chemiluminescence instrument. The red box in the figure is the imprint of the target protein and the blue imprint is the Marker.

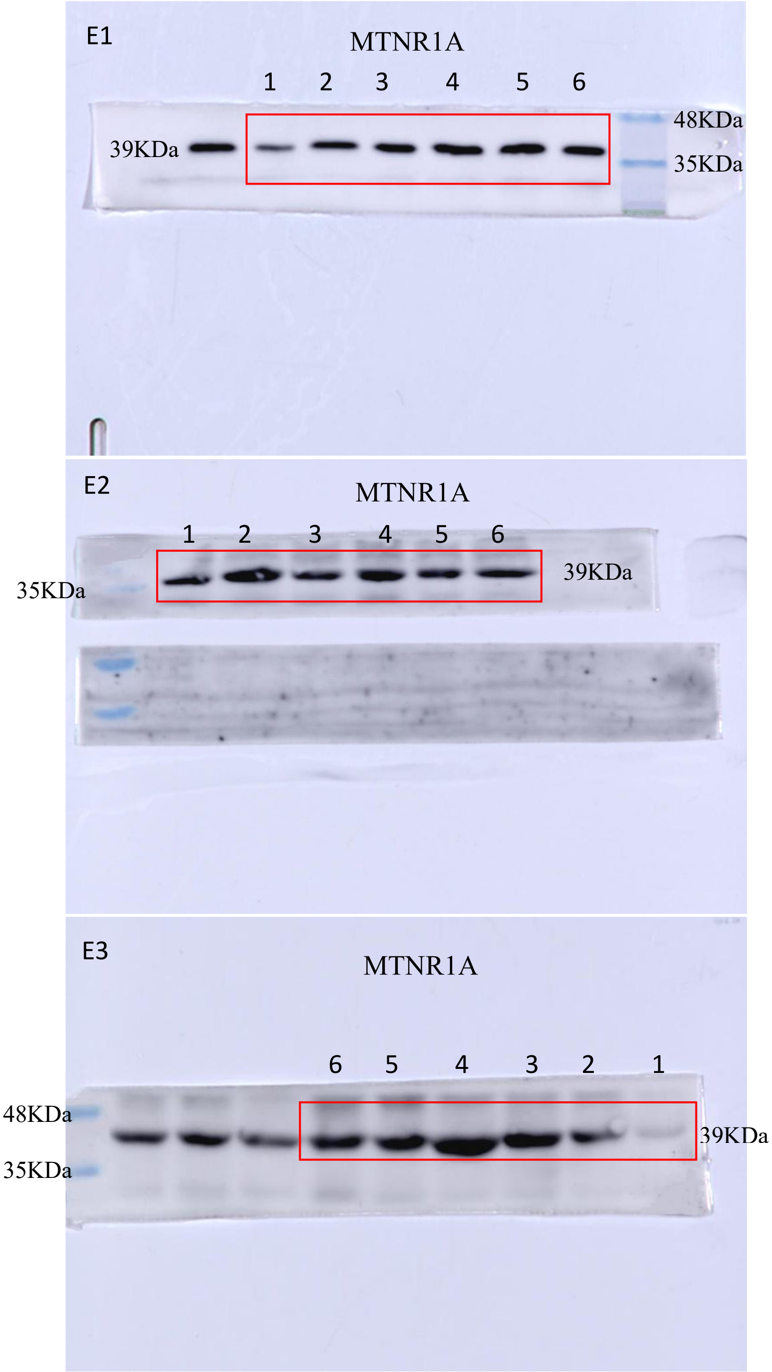

Figures E1, E2, and E3 are protein gel blots of three replicate experiments of Small Tail Han sheep MTNR1A. 1, 2, 3, 4, 5, 6 represent hypothalamus, hypophysis, testis, epididymal caput, epididymal corpus, and epididymal cauda, respectively. All Western blot gels are cropped with reference to the Marker prior to hybridization with the antibody. Gel strips are developed in a chemiluminescence instrument. The red box in the figure is the imprint of the target protein and the blue imprint is the Marker.

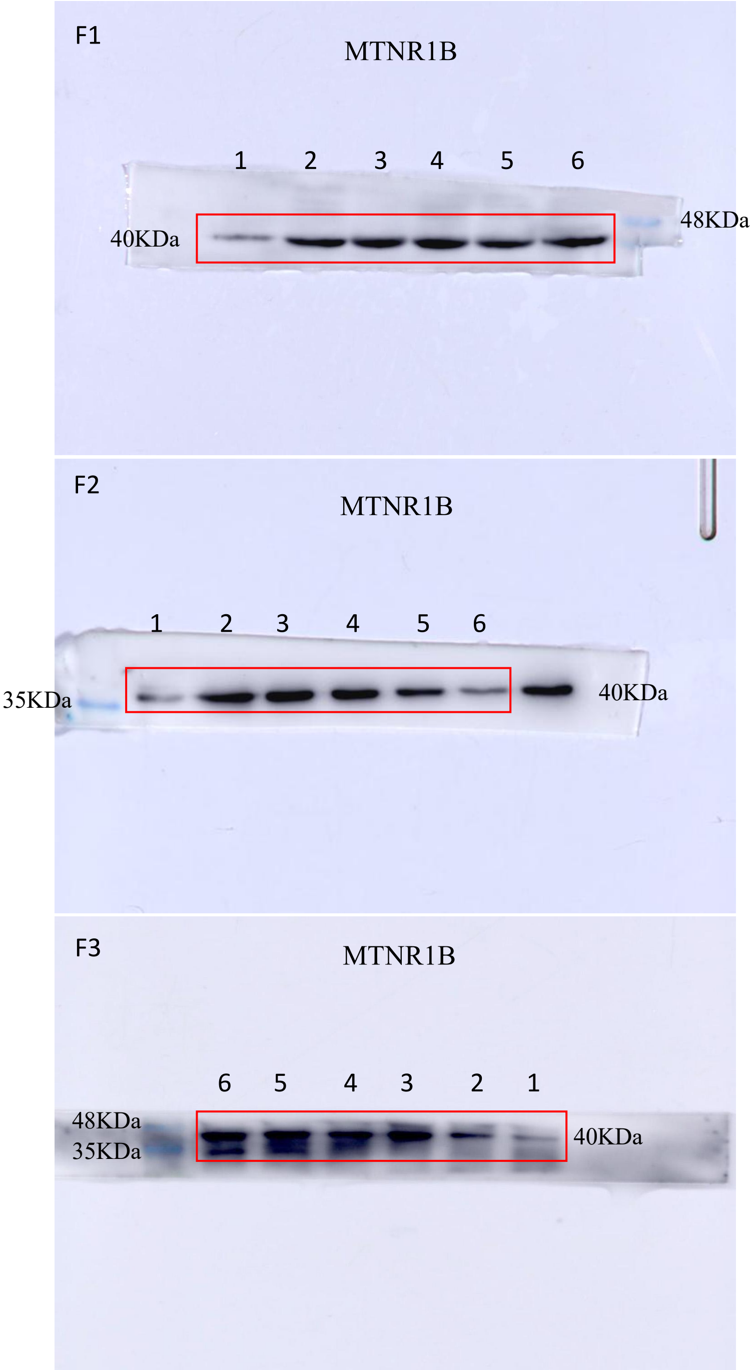

Figures F1, F2, and F3 are protein gel blots of three replicate experiments of Small Tail Han sheep MTNR1B. 1, 2, 3, 4, 5, 6 represent hypothalamus, hypophysis, testis, epididymal caput, epididymal corpus, and epididymal cauda, respectively. All Western blot gels are cropped with reference to the Marker prior to hybridization with the antibody. Gel strips are developed in a chemiluminescence instrument. The red box in the figure is the imprint of the target protein and the blue imprint is the Marker.
