# Supplementary material for: Expression and role of melatonin membrane receptors in the hypothalamic-pituitary-testicular axis of Tibetan sheep in a plateau pastoral area
Source: PLoS One. 2023 Oct 25;18(10):e0290775. doi: 10.1371/journal.pone.0290775 (PMC10599587; doi:10.1371/journal.pone.0290775)
Supplement: Attachment — Submitted filename: Comments.docx [file pone.0290775.s002.docx]

**Comments on the Evaluated Manuscript**

**PONE-D-23-06874**

**Expression and Role of Melatonin Membrane Receptors in the Hypothalamic-Pituitary-Testicular Axis of Tibetan Sheep in a Plateau Pastoral Area**

The author in the present study worked on analyzing the expression of melatonin receptors (MTNR1A and MTNR1B) in different tissues of the HPTA axis. They also presented the characteristic difference between seasonal Tibetan sheep and non-seasonal Small Tail Han sheep. The conceptualization of the study and its execution is good, but I understand the manuscript requires an overhauling major revision in terms of design of certain experiments, conclusion obtained from the present data and presentation of results.

1. The authors have examined the morphological characteristics of tissues using H and E staining and electron microscopy. The biggest concern I have about this section is the detailed elaboration of morphological cell types and features presented are insufficiently supported due to limitation of these techniques for localization specially biomarker based.

Line 237-239 *The epithelium of the epididymal duct consisted of principal cells, basal cells, halo cells, 238 pseudostratified columnar epithelium, and ciliated structures, and a large number of mature 239 spermatozoa could be observed in the epididymal duct*.

It would be difficult and rather beyond the capacity of H\E staining to differentiate the type of cells present in the epitheliums of the epididymal duct of testis tissue.

Line 243: *Scanning electron microscopy showed that microglia were pancake-shaped and uniformly distributed in Tibetan sheep hypothalamic tissue*

It’s difficult to conclude the shape of the highlighted cells marked as microglia and moreover in absence of immunolocalization its difficult to assess the type of cell.

Line 289: A. *In the hypothalamus, MTNR1A and MTNR1B were mainly distributed around microglia (Fig. 4A1 and A2)…*

Its difficult to assess the type of cells and morphological characteristics by the attached figures.

2. The author while analyzing the level of expression by immunofluorescence (Line 302) cannot compare the fluorescence intensities to evaluate expression among two different antibodies ( MTNR1A and MTNR1B). I suggest authors to remove this comparative account as many factors determine the fluorescence intensity: the affinity, avidity, clonality, types of epitopes chosen ….and stringency of washing ( if not taken into account) .

3. Mere presence of MT receptor in microglia cannot be the basis of concluding that microglia may have role in seasonal regulation of melatonin and specially when we have not even shown them by co-localization.

4. Concluding that MTNR1A and MTNR1B are the primary membrane receptors mediating the regulatory role of MT in the HPTA of sheep based on the present experiment is erroneous as no experiments were conducted to see the expression of other subtypes MTNR1C…

5. The author had present detailed comparative localization of difference in mRNA and proteins in HPTA tissues which is a good finding and may avoid morphological comparisons without solid experimental data.
