# Supplementary material for: Expression and role of melatonin membrane receptors in the hypothalamic-pituitary-testicular axis of Tibetan sheep in a plateau pastoral area
Source: PLoS One. 2023 Oct 25;18(10):e0290775. doi: 10.1371/journal.pone.0290775 (PMC10599587; doi:10.1371/journal.pone.0290775)
Supplement: Attachment — Submitted filename: Response to Reviewers 2.doc [file pone.0290775.s003.doc]

**Response to Reviewers 2**

Dear editors and reviewers,

Thank you for kindly reviewing our manuscript entitled “Expression and Role of Melatonin Membrane Receptors in the Hypothalamic-Pituitary-Testicular Axis of Tibetan Sheep in a Plateau Pastoral Area”, which we submitted to *Plos one* for publication. We greatly appreciate your detailed and valuable comments and suggestions. Here are our responses to the questions you raised.

Reviewer #2: The paper "Expression and Role of Melatonin Membrane Receptors in the Hypothalamic-Pituitary" by Yang et al. evaluated expression of MTNR1A and MTNR1B in the hypothalamic-pituitary-testicular axis (HPTA) of Tibetan sheep at different reproductive stages. The authors suggested that this study should help advance research on the mechanism of reproductive regulation of the HPTA in male animals and provide reference data for improving the reproductive rate of seasonal breeding animals. Although the question addressed in this paper could have been of interest for the scientific community, this manuscript needs of modifications.

**Point 1**:The authors should provide an adequate introduction for their manuscript. Specifically, revise the second and fourth paragraphs. They are confusing. The hypothesis and aims of the study could be clearer.

**Response 1:** Thank you for your valuable comment. Following your suggestions we have revised the second and fourth paragraphs of the manuscript and added experimental hypotheses to the manuscript (L61-63 and L91-98).

**Point 2**:For this reviewer, the Figure 1 does not improve the understanding of the text. It is suggested to delete.

**Response 2:**Thank you for your kind suggestion. We have removed Figure1 from the manuscript (L70-71).

**Point 3**:Were slaughterhouse samples used? This can raise raises questions about the nutritional status of the animals and brings more individual variability. We recommend, if possible, to provide this information. In addition, it would be appropriate to indicate the season at which the samples were collected.

**Response 3:** Thank you for your question. Our samples were collected from a breeding cooperative in Datong District, Xining City, Qinghai Province. After a health status check, we selected healthy, well-nourished, disease-free adult rams and transported them to a designated slaughterhouse, where our team of veterinarians executed the rams by carotid artery bloodletting and subsequently collected samples. We have added relevant notes in the "Materials and Methods" section of the manuscript, as well as the time of sample collection (L108-110 and L112-114).

**Point 4**:The material and methods section lacks several important methodological details; add reference for methodologies used. Although many journals seem to accept IHC results without what I and many others consider appropriate controls, this can be a concern. The absence of staining when the primary antibody is omitted is a control for nonspecific binding of the secondary antibody; this result is not evidence for the specificity of staining with the primary antibody. The proper negative control should be substitution of serum or isotype-specific immunoglobulins at the same protein concentration as the primary antibody. Was any positive control used?

**Response 4:**Thank you for your kind suggestion. We have added references to the experimental methods in the manuscript. The negative control method you described is very correct, and our negative control experiments did use the same concentration of goat serum to replace the primary antibody, we just omitted these in the description. We have made changes in the experimental methods of the manuscript (L140-141).

**Point 5**:Statistical analysis: Did the authors check the data for normal distribution?

**Response 5:** Thank you for your valuable comment. Yes, we have tested the data for normal distribution before performing the "t" test, the software we used was SPSS 17.0, and the method used was the graphical test, and we found that all the data conformed to the characteristics of the normal distribution by plotting the histograms. We have made changes in the manuscript (L229-230).

**Point 6**:I think part of the discussion needs improvement. I consider the lack of some lines that give greater importance to the work carried out, emphasizing its biological relevance and possible practical applications of the findings. Please expand on this.

**Response 6:**Thank you for your kind advice. We have revised the manuscript as required (L420-424, L445-449, L493-496, and L522-526).
